# Supplementary material for: Bayesian refinement of protein structures and ensembles against SAXS data using molecular dynamics
Source: PLoS Comput Biol. 2017 Oct 18;13(10):e1005800. doi: 10.1371/journal.pcbi.1005800 (PMC5662244; doi:10.1371/journal.pcbi.1005800)
Supplement: S1 Archive — (BZ2) [file pcbi.1005800.s011.tar.bz2 › gromacs_4.62_bayesian_saxs_refi/share/html/online/g_morph.html]

g\_morph


| g\_morph Main Table of Contents |

**VERSION 4.6  
Sat 19 Jan 2013**

---

### Description

g\_morph does a linear interpolation of conformations in order to
create intermediates. Of course these are completely unphysical, but
that you may try to justify yourself. Output is in the form of a
generic trajectory. The number of intermediates can be controlled with
the -ninterm flag. The first and last flag correspond to the way of
interpolating: 0 corresponds to input structure 1 while
1 corresponds to input structure 2.
If you specify -first < 0 or -last > 1 extrapolation will be
on the path from input structure x\_1 to x\_2. In general, the coordinates
of the intermediate x(i) out of N total intermediates correspond to:

x(i) = x\_1 + (first+(i/(N-1))\*(last-first))\*(x\_2-x\_1)

Finally the RMSD with respect to both input structures can be computed
if explicitly selected (-or option). In that case, an index file may be
read to select the group from which the RMS is computed.

### Files

| option | filename | type | description |
| --- | --- | --- | --- |
| **-f1** | conf1.gro | Input | Structure file: gro g96 pdb tpr etc. |
| **-f2** | conf2.gro | Input | Structure file: gro g96 pdb tpr etc. |
| **-o** | interm.xtc | Output | Trajectory: xtc trr trj gro g96 pdb cpt |
| **-or** | rms-interm.xvg | Output, Opt. | xvgr/xmgr file |
| **-n** | index.ndx | Input, Opt. | Index file |

### Other options

| option | type | default | description |
| --- | --- | --- | --- |
| **-[no]h** | bool | no | Print help info and quit || **-[no]version** | bool | no | Print version info and quit || **-nice** | int | 0 | Set the nicelevel || **-[no]w** | bool | no | View output .xvg, .xpm, .eps and .pdb files || **-xvg** | enum | xmgrace | xvg plot formatting: xmgrace, xmgr or none || **-ninterm** | int | 11 | Number of intermediates || **-first** | real | 0 | Corresponds to first generated structure (0 is input x\_1, see above) || **-last** | real | 1 | Corresponds to last generated structure (1 is input x\_2, see above) || **-[no]fit** | bool | yes | Do a least squares fit of the second to the first structure before interpolating |

---

http://www.gromacs.org  
gromacs@gromacs.org
